# Open-gate mutants of the mammalian proteasome show enhanced ubiquitin-conjugate degradation

**DOI:** 10.1038/ncomms10963

**Published:** 2016-03-09

**Authors:** Won Hoon Choi, Stefanie A. H. de Poot, Jung Hoon Lee, Ji Hyeon Kim, Dong Hoon Han, Yun Kyung Kim, Daniel Finley, Min Jae Lee

**Affiliations:** 1 Department of Biomedical Sciences, Seoul National University Graduate School, Seoul 03080, Korea; 2 Department of Biochemistry and Molecular Biology, Seoul National University College of Medicine, Seoul 03080, Korea; 3 Department of Cell Biology, Harvard Medical School, Boston, Massachusetts 02115, USA; 4 Center for Neuro-Medicine, Korea Institute of Science and Technology (KIST), Seoul 02790, Korea; 5 Neuroscience Research Institute, Seoul National University College of Medicine, Seoul 03080, Korea

## Abstract

When in the closed form, the substrate translocation channel of the proteasome core particle (CP) is blocked by the convergent N termini of α-subunits. To probe the role of channel gating in mammalian proteasomes, we deleted the N-terminal tail of α3; the resulting α3ΔN proteasomes are intact but hyperactive in the hydrolysis of fluorogenic peptide substrates and the degradation of polyubiquitinated proteins. Cells expressing the hyperactive proteasomes show markedly elevated degradation of many established proteasome substrates and resistance to oxidative stress. Multiplexed quantitative proteomics revealed ∼200 proteins with reduced levels in the mutant cells. Potentially toxic proteins such as tau exhibit reduced accumulation and aggregate formation. These data demonstrate that the CP gate is a key negative regulator of proteasome function in mammals, and that opening the CP gate may be an effective strategy to increase proteasome activity and reduce levels of toxic proteins in cells.

## Abstract (web summary)

The proteasome plays a key role in proteostasis by mediating the degradation of ubiquitinated substrates. Here the authors show that an open-gate mutant of the proteasome is hyperactive towards a subset of substrates and can effectively delay the accumulation of toxic protein aggregates.

## Graphical abstract

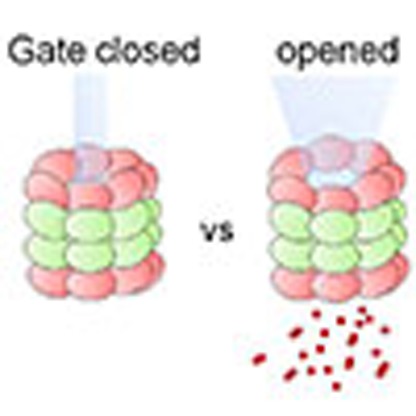

The 26S proteasome, a ∼2.5-MDa holoenzyme complex, is the sole adenosine triphosphate (ATP)-dependent protease in the eukaryotic cytosol and nucleus, and mediates the irreversible degradation of target substrates conjugated to ubiquitin. It controls intracellular protein levels on a global scale and in particular plays a key role in protein quality control^1^,^2^. The proteasome holoenzyme (or 26S proteasome) comprises of the 28-subunit core particle (CP, also known as the 20S) and the 19-subunit regulatory particle (RP, also known as the 19S or PA700)^3^. At the interface between the RP and CP, two ring assemblies are axially aligned: the heterohexameric ATPase ring of the RP (known as the RPT ring, and composed of RPT1-RPT6) and the heteroheptameric α-ring of the CP (composed of α1–α7). A number of reversibly associated proteins have been identified, some of which influence the activity of proteasomes^4^,^5^,^6^. The overall architecture of the proteasome was recently established through cryo-electron microscopy studies^7^,^8^.

The CP is composed of four heteroheptameric rings, thus forming an α_7_β_7_β_7_α_7_ structure. The outer rings of α-subunits form the substrate translocation channel while the β-subunit-forming inner rings contain six proteolytic active sites (two trypsin-like, two chymotrypsin-like and two caspase-like, in specificity) in their interiors. ATP-dependent protease complexes typically have proteolytic sites sequestered within CP-like cylinders^9^. Broad-spectrum proteasome inhibitors, such as bortezomib, target these sites, and are effective anti-cancer agents^10^. The RP interacts with the polyubiquitin chains of the substrate and translocates the substrates into the CP, with substrate deubiquitination occurring either prior to or contemporaneously with translocation^7^. Deubiquitination on the RP may promote or delay proteasomal degradation, possibly depending on the coordination between the rates of ubiquitin chain trimming and substrate translocation^11^,^12^,^13^,^14^,^15^. Due to the exceptional complexity of the system, many of the regulatory mechanisms of proteasome activity and homoeostasis remain to be elucidated.

In the free CP (CP that is not engaged with the RP), the N-terminal tails of the α-subunits fill the centre of the ring. They are tightly interlaced to form the gate, blocking substrate access into the proteolytic chamber^16^,^17^. On binding of the RP, the N-terminal tails are displaced, removing the block to substrate translocation. Gate opening is driven by docking of the C-terminal tails of a subset of RPT proteins into the seven intersubunit pockets of the α-subunits^18^. In addition to the RP, other endogenous activators of the CP gate include proteasome activator 28αβ (PA28αβ, also known as the 11S), PA28γ, PA200/Blm10 (ref. 1). The RPT ring creates the RP substrate translocation channel that is then attached to the CP channel^7^. A tight co-alignment of the RP and CP channels is generated by conformational change when the proteasome is engaged with polyubiquitinated substrates or ATPγS^19^,^20^. ATP-driven conformational dynamics of the RPT ring induce substrate translocation and unfolding probably through either concerted or sequential programs of ATP hydrolysis around the ring^21^,^22^.

Previous studies using the yeast proteasome indicated that, among the key components of the gate, such as α2, α3 and α4, deletion of the N-terminal tail of the α3 subunit resulted in conformational destabilization of other N-terminal residues and consequently opening of the CP channel into the proteolytically active interior chamber^16^,^23^. Substrate translocation channels and the regulated gates into the proteolytic sites might be a general theme for ATP-dependent proteases. However, the gating of mammalian proteasomes and the consequences of gate opening in mammalian cells are essentially uncharacterized.

To understand the role of the CP gate in mammalian proteasomes, we generated human cell lines that stably express α3ΔN subunits. We observed enhanced activity of purified mutant proteasomes measured by hydrolysis of fluorogenic peptides and degradation of polyubiquitinated protein substrates. The hyperactivity of α3ΔN proteasomes was observed for both free CP and holoenzyme complexes. We also found that the increased cellular proteasome activity of α3ΔN proteasomes stimulated substrate degradation and significantly delayed tau aggregate formation in cultured cells. Finally, multiplexed quantitative proteomics using isobaric tandem mass tags (TMTs) revealed that levels of ∼200 proteins were significantly reduced in the α3ΔN cells. These findings indicate the importance of the regulated CP channel in mammals, which functions as a rate-limiting step in proteasome-mediated proteolysis, and suggest that α3ΔN proteasomes could potentially help cells to cope with the proteotoxic stresses implicated in various neurodegenerative diseases.

## Results

### Generating open-gated mutant proteasomes

**Figure 1:**
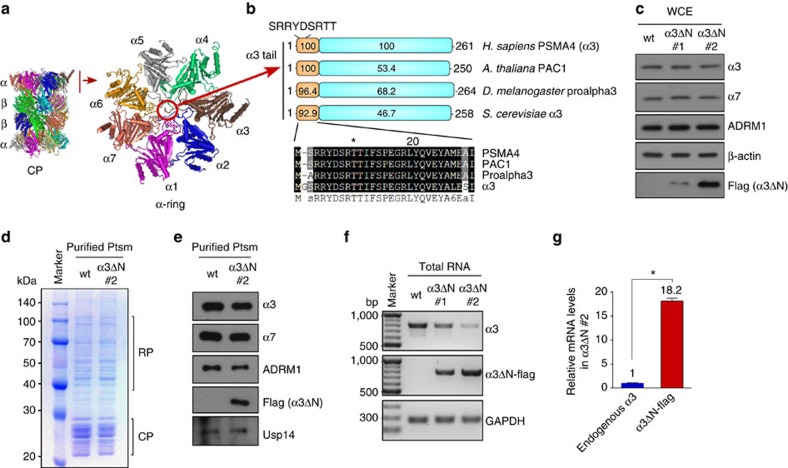
Generation of the open-gated mammalian proteasome (α3ΔN proteasome) through deletion of the α3 tail. (**a**) A side view of the proteasome core particle (CP) with an α_7_β_7_β_7_α_7_-stacked ring structure and a top view of the α-ring (PDB ID: 1IRU). The protruding nine N-terminal residues (SRRYDSRTT) of human α3 (indicated by the red circle) were deleted to generate the open-gated mutant of human proteasomes. (**b**) Alignment of the highly conserved N-terminal regions of α3 orthologs from yeast α3 to human PSMA4, which function as gates of the CP. Relative identity scores for the aligned N-terminal regions and the remaining regions of α3S are shown. Residues invariant or conservatively replaced in at least 75% of the sequences are shown on black or grey backgrounds, respectively. (**c**) α3ΔN cell lines that stably express biotin tags at the β4 subunit of proteasomes were generated through transient overexpression of the mutant proteasome and subsequent selection of dominant negative clones. Whole cell extracts (WCEs) from two clones (α3ΔN clone #1 and #2) were analysed by immunoblotting (IB) assay. Wild-type (wt) indicates the parental 293-β4-biotin cell line. Signals from the flag IB indicate overexpressed mutant α3ΔN. (**d**) Comparison of purified proteasomes (Ptsm), which indicates that α3ΔN proteasomes have no distinct composition changes. Coomassie-stained SDS–PAGE gel. (**e**) Same as **d**, except IB analysis was performed using various antibodies against CP and RP subunits, and flag for α3ΔN. (**f**) mRNA levels of endogenous α3 and overexpressed α3ΔN measured by reverse transcription–polymerase chain reaction (RT–PCR). (**g**) Same as **f**, except quantitative RT–PCR (qRT–PCR) was used to compare α3 mRNA levels in the α3ΔN #2 cell line, which determined that ∼18 times more α3ΔN subunits were expressed compared with endogenous α3. *P<0.001 (*n*=3, two tailed Student's *t*-test).

Of the seven α-tails, that of α3 projects most deeply into the centre of the translocation channel, at the same time contacting and potentially stabilizing the N-terminal tails of many other α-subunits (Fig. 1a). In addition, this region is evolutionarily conserved across the eukaryotes (for example, 92.9% identity between humans and yeast α3 N-termini) to a high degree, in contrast to the body of α3, which is less than 50% identical between humans and yeast (Fig. 1b). The virtually complete conservation of α3 N-termini suggests a common gating mode for the CP channel from yeast to mammals. To study gating of the substrate translocation channel in mammals, we stably overexpressed a flag-tagged form of α3 with a 9-residue deletion encompassing the tail element. Overexpression was carried out in the HEK293-β4-biotin cell line that allows for rapid purification of human proteasomes, either 20S and 26S forms, via the β4 subunit of the CP^24^. Two clones of stable cell lines that expressed different amounts of exogenous α3ΔN-flag were obtained, with the α3ΔΝ #2 clone (hereafter referred to as the α3ΔΝ cell line) showing the more prominent expression of the mutant subunit (Fig. 1c).

Active human 26S proteasomes were affinity-purified from the parental (wild type) and α3ΔN cells (Fig. 1d,e). The overall integrity and abundance of α3ΔN proteasome holoenzymes were virtually identical to those of wild type. In addition, the stoichiometry of α3 within the proteasome appeared to be proper in the mutant complex. Fortuitously, endogenous α3 mRNA expression was dramatically downregulated on α3ΔΝ-flag mRNA expression (Fig. 1f). Quantitative RT–PCR using primers specific for either endogenous and exogenous α3s indicated that the mutant α3ΔΝ mRNA levels were ∼18 times higher than the endogenous α3 mRNA (Fig. 1g), suggesting that the stable α3ΔΝ cell line had predominantly open-gated proteasomes. At this ratio of mutant to wild type, CP from α3ΔN cells would be expected to exhibit wild-type gating in less than 1 of 300 complexes. Importantly, the total cellular level of α3 was comparable between the α3ΔN #2 clone and the parental cell line.

### Enzymatic properties of α3ΔN mammalian proteasomes

**Figure 2:**
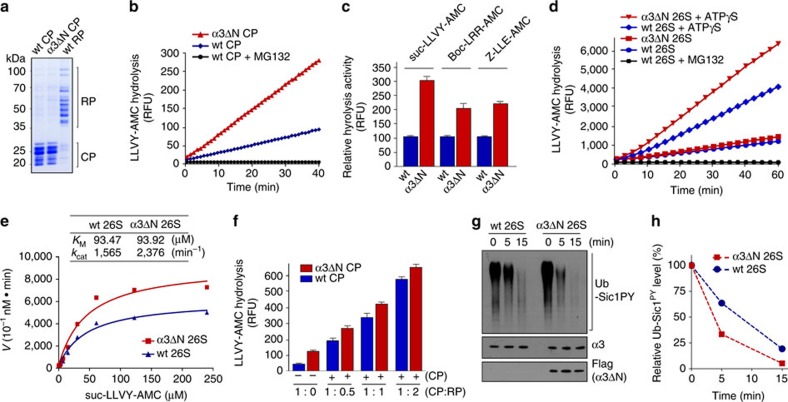
α3ΔN proteasomes showed enhanced proteolytic activity compared with wild-type proteasomes. (**a**) Purified CP and RP from the wt and α3ΔN cell lines. (**b**) suc-LLVY-AMC assay using purified CP. α3ΔN CP showed ∼three-fold higher suc-LLVY-AMC hydrolysis activity than wt CP. (**c**) The three different proteolytic sites in the purified CP were measured by using fluorogenic peptide substrates suc-LLVY-AMC (for chymotrypsin-like activity), Boc-LRR-AMC (for trypsin-like) and Z-LLE-AMC (caspase-like). (**d**) Same as **b**, except 26S proteasomes were used to measure suc-LLVY-AMC hydrolysis activity in the presence and absence of ATPγS, a slowly hydrolyzable analogue of ATP. (**e**) Michaelis–Menten plot, *K*_M_, and *k*_cat_ values of wt and α3ΔN 26S proteasomes with ATPγS on concentration-dependent suc-LLVY-AMC cleavage for 15 min. The data were fit to a hyperbolic curve by nonlinear regression (*R*^2^>0.98) to calculate the enzyme kinetic data. The graphs shown are representative of at least three independent determinations and each data point is the mean±s.d. (**f**) Reconstitution of the holoenzymes using wt RP and wt or α3ΔN CP in various molar ratios. (**g**) Ub-Sic1^PY^ degradation assay using wt and α3ΔN 26S proteasomes. Reactions incubated with Ub-Sic1^PY^ and purified proteasomes for the indicated times were analysed by SDS–PAGE/IB using anti-T7 for Sic1, anti-α3 and anti-flag antibodies. (**h**) Quantification of Ub-Sic1^PY^ proteins in the degradation assay.

We isolated the CP from α3ΔΝ cells (Fig. 2a) and found significantly elevated activity compared with wild-type CP, as measured by suc-LLVY-AMC hydrolysis, which is specific for the chymotrypsin-like β5 activity (Fig. 2b). The trypsin-like β2 and caspase-like β1 activities were similarly elevated, measured by Boc-LRR-AMC and Z-LLE-AMC hydrolysis, respectively (Fig. 2c). The parallel effects on all proteolytic sites indicates that the hyperactivity of mutant proteasomes reflects CP gate opening rather than allosteric modulation of active sites in the catalytic chamber.

The hyperactivity of the open-gated CP was also observed when the CP forms the 26S holoenzyme with the RP, especially in the presence of ATPγS, a slowly hydrolyzable analogue of ATP. ATP binding, not ATP hydrolysis, is thought to be sufficient to promote the 26S proteasome assembly from RP and CP, and substrate translocation^25^,^26^. Under the ATPγS-enriched conditions, the RP undergoes significant intersubunit rearrangement from a pre-engaged conformation to an engaged conformation, which exhibits coaxial alignment between translocation channels of the RPT ring and the α-ring^19^,^20^,^27^. This conformation is similar to that of 26S proteasomes when they are in translocation-competent state when associated with polyubiquitinated substrates^20^,^27^. Consistent with previous findings^25^,^26^,^28^, the peptide hydrolysis activity of wild-type 26S proteasomes was significantly stimulated in the presence of ATPγS (Fig. 2d). This activity stimulation by ATPγS was more dramatic on the α3ΔΝ 26S proteasome, which showed ∼1.6 times higher peptidase activity than wild type (Fig. 2d).

Using suc-LLVY-AMC, we measured the enzyme kinetics of translocation-competent 26S proteasomes. The *k*_cat_ value of α3ΔΝ 26S (2,376 min^−1^) was significantly higher than that of wild-type 26S (1,565 min^−1^) while *K*_M_ values were comparable (93.92 μM for α3ΔN versus 93.47 μM for wild type) (Fig. 2e). These kinetic data indicate that the deletion of the N-terminal tail of α3 mainly affects substrate entry rather than the proteolytic sites of the CP. When they were in the non-engaged conformations or in the presence of ATP, α3ΔΝ holoenzymes showed only modestly enhanced proteolytic activity (Fig. 2d). In addition, the peptidase activity of both the wild type (∼10-fold when CP:RP molar ratio was 1:2) and α3ΔΝ CP (∼5-fold) was significantly stimulated when complexed with purified RP (Fig. 2f). By reconstituting purified CP and RP with different molar ratios, we identified maximum stimulation when the molar ratio of CP and RP was 1:2. Similar to the results obtained using purified 26S proteasomes, the reconstituted CP–RP complex showed only modestly increased proteasome activity with α3ΔN CP in comparison with wild-type CP. However, as shown above, RP stimulation is not sufficient for the proteasomes to achieve their fully activated status required for efficient substrate degradation (Fig. 2d; Supplementary Fig. 1). Our data imply that gate opening by the RP may be incomplete, and that the α3 tail is critical for the residual occlusive effect of the gate in the holoenzyme state.

We next examined whether the open-gated mutant proteasome has enhanced proteolytic activity using a more physiologically relevant protein substrate, polyubiquitinated Sic1^PY^ (Ub-Sic1^PY^), a CDK inhibitor from *Saccharomyces cerevisiae*, instead of fluorogenic peptide substrates. A modified form of Sic1, in which the PY element signals polyubiquitination with mixed Ub-linkage types, was employed in these *in vitro* degradation assays^29^. The purified α3ΔΝ 26S proteasomes showed more rapid degradation of Ub-Sic1^PY^ than wild-type proteasomes (Fig. 2g,h). Thus, opening the central gate of the CP in the mammalian proteasome promotes degradation of protein substrates when the RP is bound to the CP. Furthermore, the facilitated degradation of Ub-Sic1^PY^ substrates by α3ΔΝ holoenzymes may reflect a more fully open state of the CP channel as revealed by the peptide hydrolysis data.

The enhancement of α3ΔΝ CP activity and the maintenance of higher activity as the 26S proteasome with engaged conformations suggests that substrate proteolysis in the catalytic core is not only CP gate-dependent, but also closely linked with other regulatory mechanisms on the proteasome. To investigate whether there are additional layers of activation required for proteasomal degradation, we tested whether blocking ATP hydrolysis influenced the proteasomal degradation of Ub-Sic1^PY^. Addition of excess ATPγS in the *in vitro* degradation assay significantly delayed the degradation of Ub-Sic1^PY^ by both wild type and α3ΔΝ 26S proteasomes (Supplementary Fig. 2), probably due to the loss of substrate translocation functionality. However, α3ΔΝ 26S proteasomes showed still facilitated Ub-Sic1^PY^ degradation and inhibited polyubiquitin chain trimming on the proteasome (Supplementary Fig. 2). The constitutive opening of the CP gate probably does not affect the substrate translocation function of the RPT ring. Thus the enhanced proteolytic capacity of mutant 26S proteasome might originate from the facilitated substrate entry rate (and possible product release as well) through the opened gate of proteasomes with engaged conformations. Taken together, our data suggest that the open-gate mutation enhances the activity of not only free CP but of proteasome holoenzyme as well. Gate opening appeared to be a regulated process even in the assembled holoenzyme, being subject to control by nucleotide and most likely substrate occupancy, and aspects of this control remain in place in the α3ΔΝ mutant. These results predict that the α3ΔΝ mutation should accelerate the degradation of ubiquitinated substrates of the proteasome in living cells, which was borne out as described below.

### Open-gated proteasomes facilitate substrate degradation in cells

**Figure 3:**
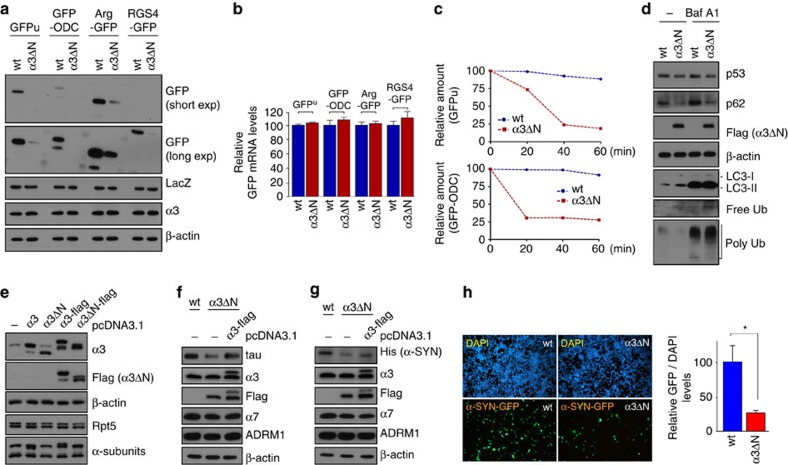
α3ΔN proteasomes showed enhanced substrate degradation in mammalian cells. (**a**) Proteasome substrates and LacZ control proteins were coexpressed in wt and α3ΔN cell lines and their levels were compared. Short exp and long exp, short and long exposure of the blot, respectively. (**b**) Same as **a**, except qRT–PCR was performed using primers for GFP and GAPDH (control for normalization). The values plotted are means±s.d. of three independent experiments (*n*=3). (**c**) The ubiquitin-dependent proteasome substrate GFPu and the ubiquitin-independent substrate GFP-ODC proteins were transiently overexpressed in wt and α3ΔN cell lines. Then chase experiments (Supplementary Fig. 3) were carried out and their quantification at the indicated time points are shown. The GFP signals were normalized to those of endogenous β-actin. (**d**) Various endogenous proteins in wt and α3ΔN cell lines were compared in the absence and presence of bafilomycin A1 (BafA1). (**e**) Constructs expressing wt α3 and α3ΔN in HeLa. (**f**) Coexpression of tau and α3-flag in α3ΔN cell lines. (**g**) Same as **f**, except α-synuclein (α-SYN) was overexpressed instead of tau. (**h**) (Left) Fluorescent microscope images of wt and α3ΔN cell lines after transiently overexpressed α-SYN-GFP. (Right) Quantification of α-SYN-GFP signals normalized to counterstained DAPI signals. Bars represent the means of percent values (relative to the GFP signal in wt cells)±s.d. from three independent experiments. *P<0.001 (*n*=3, two-tailed Student's *t*-test).

The results above indicated that the α3ΔN proteasomes, both free CP and holoenzyme complexes, have significantly enhanced proteolytic activity. The effects of gate-opening in living cells were then investigated using α3ΔΝ cells. The steady-state levels of various transiently overexpressed proteasome substrates, including GFP^U^ (a Ub-dependent substrate), GFP-ODC (Ub independent), Arg-GFP and RGS4-GFP (two Ub-dependent N-end rule substrates), were significantly lower in the α3ΔΝ cell line, while levels of cotransfected lacZ were comparable (Fig. 3a). The GFP mRNA levels were also not changed in the mutant cell line in the presence of all substrates (Fig. 3b), indicating that the reduction in model substrate levels is post-translational. Chase experiments were performed after release from short-term MG132 treatment because of the rapid turn-over rates of these substrates. The result confirmed facilitated degradation of GFP^U^ and GFP-ODC in the α3ΔΝ cells (Fig. 3c). The GFP^u^ and GFP-ODC protein levels in two cell lines were comparable after MG132 treatment, further indicating their accelerated degradation by the hyperactive mutant proteasome (Supplementary Fig. 3). Moreover, among the substrates, GFP-ODC, a Ub-independent proteasome substrate, was more responsive to the gate-opening mutation (Supplementary Fig. 3). This dramatic effect in cultured cells may reflect the fact that the proteasome exists as free CP, RP–CP (singly capped) and RP_2_–CP (doubly capped proteasomes) forms in the cell and that ODC proteins are degraded by both free CP and holoenzyme complexes^30^.

Enhancing proteasome activity in the cell also resulted in reduced levels of the cell cycle checkpoint protein p53 and the selective autophagy receptor p62, which were accompanied by increased free (unconjugated) Ub and decreased polyubiquitin levels (Fig. 3d). The conjugated forms of Ub are expected to be more sensitive to proteasome activity than free forms^31^,^32^. We also observed increased LC3-II levels in the α3ΔΝ cells compared with wild-type cells, but this effect was lost when bafilomycin A1, an inhibitor of the late stage of autophagy, was used (Fig. 3d). These findings suggested that the autophagic flux was inhibited at the autophagosome–lysosome fusion step when cellular proteasome activity was enhanced. Consistent with this, a significantly increased number of GFP-LC3 puncta were observed in the hyperactive α3ΔΝ cells (Supplementary Fig. 4). Therefore, the dynamic activity regulation between the ubiquitin–proteasome system (UPS) and the autophagy–lysosome system appears to be linked through the proteasome activity.

We then examined the degradation of various proteotoxic proteins including tau and α-synuclein (α-Syn), which are implicated in Alzheimer's and Parkinson's diseases, respectively, when accumulated and aggregated^33^,^34^. Both of these proteins are substrates of the proteasome and impaired proteasomal activity may be related to the progression of these diseases^35^. In the α3ΔΝ cells, levels of both overexpressed tau and α-Syn were dramatically decreased compared with those in control cells (Fig. 3e–g; Supplementary Fig. 5). This outcome is also likely contributed by both the open-gated CP and holoenzyme complexes because the CP is known to degrade intrinsically unstructured proteins, including tau and α-Syn^36^. However, adding back wild-type α3 to the mutant cells effectively abrogated the CP gate-opening effect by α3ΔΝ on tau and α-Syn degradation (Fig. 3f,g). Moreover, the hyperactivity of proteasomes in the α3ΔΝ cells significantly delayed the formation of α-Syn aggregates (Fig. 3h), which might be preceded by the accelerated degradation of soluble α-Syn^37^. No effects on α-Syn or tau mRNA level were observed either as a consequence of α3ΔΝ mutation or by rescuing wild-type α3 (Supplementary Fig. 6), further indicating that facilitated proteasomal degradation results in the decreased levels of these proteins in mammalian cells.

### Enhanced tau degradation by open-gated proteasomes

**Figure 4:**
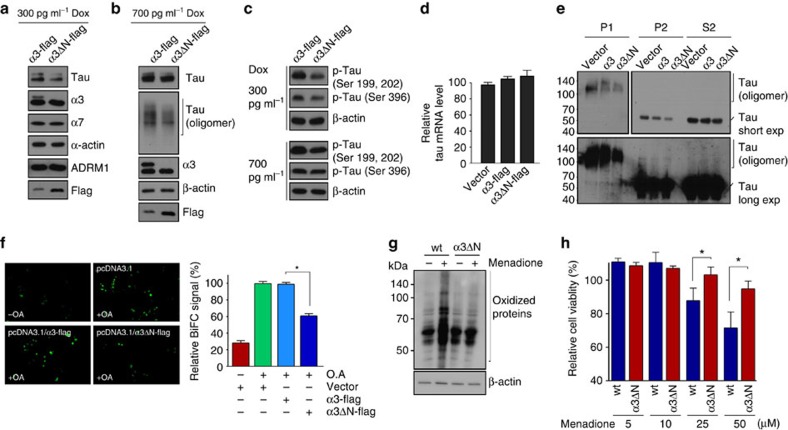
Facilitated tau protein degradation and delayed tau aggregation by α3ΔN gate opening. (**a**) Total tau proteins were detected after expression of wt α3 and α3ΔN in the inducible tau cell line treated with 300 pg ml^−1^ doxycycline (Dox). (**b**) Same as **a**, except that 700 pg ml^−1^ Dox treatment was used to detect aggregated forms of tau. When α3ΔN was coexpressed, levels of tau oligomers were significantly decreased. (**c**) Inducible tau cell lines were treated with 300 pg ml^−1^ (upper panel) and 700 pg ml^−1^ Dox (lower), indicating α3ΔN gate opening enhances the degradation of phosphorylated tau proteins as well. Samples were analysed by immunoblotting using phosphorylation-specific tau antibodies (Ser 396 and Ser 199/202). (**d**) qRT–PCR to compare tau mRNA levels in the inducible tau cell lines after transfection and 300 pg ml^−1^ Dox treatment. (**e**) Same as **a**, except tau was induced by 500 ng ml^−1^ Dox, and tau in SDS-soluble and -insoluble fractions were separately isolated and compared (see Methods). P1, P2 and S2 denote pellets after 200*g*, pellets after 16,000*g* and supernatants after 16,000*g* centrifugation runs, respectively, using Triton X-100-based lysis buffer. Short exp and long exp, short and long exposure of the blot, respectively. (**f**) Comparison of tau oligomerization after tau-BiFC cell lines were transfected with α3 or α3ΔN. Values represent the mean (±s.d.) of three independent cultures including a total of ∼10,000 cells. OA, okadaic acid. (**g**) Wild-type (wt) and α3ΔN cell lines were treated with menadione (25 μM) for 2 h. Oxidized proteins in whole-cell lysates were labelled with 2-4-dinitrophenyl hydrazine (DNPH), and immunoblotting with anti-dinitrophenyl (DNP) antibody was performed. (**h**) Cell survival under oxidative stress was measured using wt and α3ΔN cells. Menadione was treated as the indicated concentrations for 4 h. Values are represented as mean±s.d. (*n*=3). *P<0.01 (one-way analysis of variance (ANOVA) with Bonferroni's multiple comparison test).

Enhanced proteasome activity may be beneficial to cells by delaying proteotoxic protein accumulation and aggregation. Tau is thought to undergo degradation via the UPS, especially during the early stages of tauopathy and Alzheimer's disease progression^35^. We used a HEK293-derived cell line that expresses the longest isoform of human tau (htau40) on doxycycline (Dox) induction (an inducible tau cell line)^38^. These cells expressed htau40 in a tightly dose-dependent manner^24^ and produced SDS-resistant tau aggregates, a pathological hallmark of AD, after ∼2 days with a high dose of Dox in culture (Fig. 4b,e). When α3ΔΝ was transfected to cells treated with 300 pg ml^−1^ Dox, the levels of induced tau proteins were mildly decreased compared with that of α3 transfection (Fig. 4a), although this effect was weaker than that of stable open-gated α3ΔΝ expression (Fig. 3f). When tau was induced with 700 pg ml^−1^ Dox, significantly reduced amounts of tau oligomers were observed (Fig. 4b). We observed weak effects of α3ΔN overexpression on monomeric tau degradation when this Dox concentration was used. Therefore, it appears that the overall levels of induced tau limit the effect of hyperactive proteasomes in mammalian cells and consequently its propensity to aggregate.

Phosphorylated tau forms intraneuronal filamentous oligomers called paired helical filaments, which are the principle constituent of neurofibrillary tangles in Alzheimer's diseases. Levels of tau proteins phosphorylated at Ser^396^ or Ser^199/202^ were also significantly reduced in 300 pg ml^−1^ Dox-treated α3ΔΝ cells, and mildly reduced in 700 pg ml^−1^ Dox conditions (Fig. 4c). Under those conditions, tau mRNA levels between wild-type and α3ΔΝ cells were virtually identical (Fig. 4d), indicating that accelerated tau degradation occurs at the post-translational stage by hyperactive proteasomes. Considering that neurodegeneration and cognitive dysfunction are critically linked to the accumulated tau level in neurons, these results indicate that enhancing proteasome activity using the open-gated proteasome could be an effective therapeutic strategies for Alzheimer's and other related neurodegenerative diseases.

Tau aggregates was further examined by separating the Triton X-100 insoluble fraction from the tau cell line induced with 700 pg ml^−1^ Dox. Consistently, we found significantly reduced levels of pelleted insoluble tau monomers and tau aggregates in the 16,000*g* (P2) and the 200*g* (P1) centrifugation runs, respectively (Fig. 4e). To visualize and quantify tau oligomerization in living cells, we utilized a htau40-expressing cell line with the biomolecular fluorescence complementation system (a tau-BiFC cell line)^39^, where fluorescence becomes strongly ‘turned-on' on tau oligomerization. Consistent with inducible tau cells, tau-BiFC cells overexpressing open-gated α3ΔΝ proteasomes showed significantly less tau aggregation compared with cells expressing α3 (Fig. 4f).

Next, we directly delivered the purified open-gated proteasomes into inducible tau cells using silica-based mesoporous nanoparticles. The nanoparticles had pore sizes between 25 and 30 nm and nickel (Ni^2+^) moieties, which enabled them to harbour a proteasome holoenzyme molecule through noncovalent interactions with the poly-histidine tag of proteasomes^37^. The levels of induced tau decreased more significantly after direct delivery of hyperactive mutant proteasomes than wild-type proteasomes (Supplementary Fig. 7), indicating that exogenous α3ΔN proteasomes delivered using nanoparticles can delay the aggregation process of tau proteins in proteotoxic conditions. Again, the magnitude of tau depression on enhancement of proteasome activity appeared to be partially dependent on the total tau levels in cells (Fig. 4a–c). These results suggest that hyperactive proteasomes may more efficiently degrade protein substrates that impose an unusual load on the UPS, such as overexpressed tau.

Next, we examined the effect of CP gate-opening on degradation of oxidized proteins, which are an important subset of misfolded substrates of proteasomes and accumulated with age. After reactive oxygen species (ROS) was induced by menadione, oxidized proteins were labelled with 2,4-dinitrophenylhydrazine, and visualized through their carbonyl group modification. The α3ΔΝ cells showed strikingly reduced levels of oxidized proteins compared with wild type after the treatment of menadione (Fig. 4g), suggesting that hyperactive proteasomes may have accelerated oxidized proteins clearance in cells. In addition, α3ΔΝ cells showed significant resistance to cytotoxicity from menadione-mediated oxidative stress (Fig. 4h). Consequences of protein aggregates in neurons include excessive generation of free radical and oxidatively damaged proteins, which are also closely linked to neuronal dysfunction and death^40^. Our results indicate that enhancing proteasome activity through opening of the CP gate might be beneficial in protecting cells under oxidative stress conditions during neurodegeneration.

### TMT-MS-based identification of α3ΔN proteasome targets

**Figure 5:**
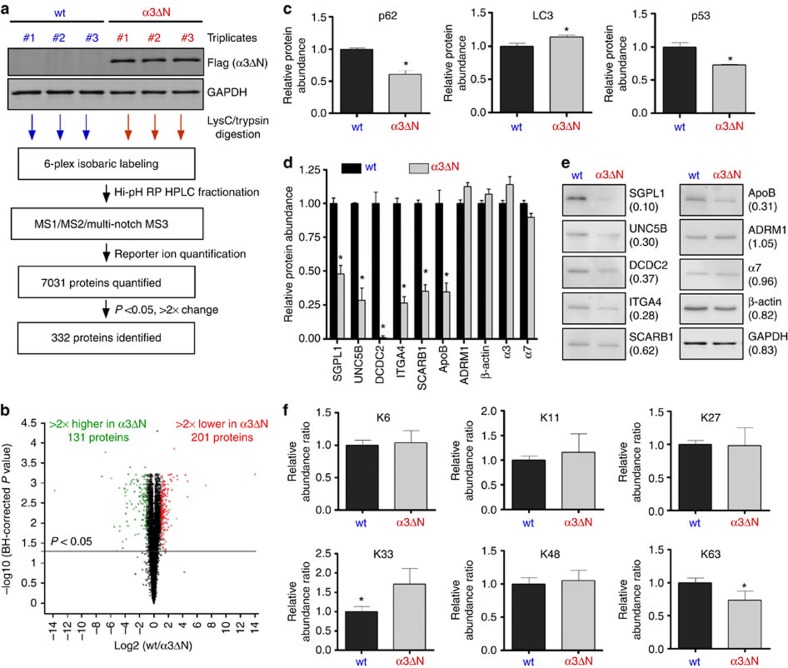
Identification and validation of changes in protein levels in α3ΔN cells. (**a**) Overview of the quantitative TMT approach used to identify the hyperactive proteasome-sensitive targets. Triplicates of whole-cell lysates from wt and α3ΔN cells were individually labelled with 6-plex isobaric tags, mixed, and analysed by LC-MS^3^. (**b**) Volcano plot of the 7,031 quantified proteins. Log2 ratios of wt/α3ΔN cells are shown with black (<2 × change), green (>2 × increase in α3ΔN) and red (>2 × decrease in α3ΔN) dots. A threshold using a *P* value cutoff (*P*=0.05) is shown in a black line. (**c**) Representative TMS-MS^3^ data, quantifying and comparing p62, LC3 and p53 levels in wt and α3ΔN cells. These data, shown here as relative abundance ratios±s.d., are consistent with the results of IB-based analysis in Fig. 3d. (**d**) Hyperactive proteasome-sensitive target substrates, whose functions are in cell motion, were quantified by TMT-MS^3^. (**e**) Immunoblotting analysis of target substrates in **d**. Quantitative ratios (wild type versus α3ΔN) are shown in parentheses. (**f**) Relative abundance ratios of specific linkage types of polyubiquitin chain from TMT-MS analysis. *P<0.05 (*n*=3, two-tailed Student's *t*-test).

The global effects of enhanced proteasome activity in mammalian cells were characterized by multiplexed quantitative proteomics based on tandem mass tags-mass spectrometry (TMT-MS) (Fig. 5a). To date, many proteomic strategies aimed at identifying proteasome substrates and ubiquitination profiles using proteasome inhibitors^41^,^42^, but a quantitative study of the UPS proteome in response to activation of the proteasome has been unavailable. Protein samples were obtained from three independent cultures of wild-type and hyperactive α3ΔN cells, which showed excellent reproducibility evaluated by the intra-group component analysis and hierarchical clustering. The six samples were independently labelled with 6-plex isobaric TMT reagents, pooled for parallel comparison, fractionated using basic RP-HPLC, and analysed using MS^3^ methods to quantify a total of 7,031 proteins (Fig. 5a; Supplementary Table 1). The initial threshold for data evaluation was a more than two-fold increase or decrease with a *P* value <0.05. By these criteria, 332 proteins showed significant changes (Fig. 5b; Supplementary Table 2). Among these responding proteins, 201 were depleted in α3ΔN cells, many of which presumably due to accelerated protein degradation via the proteasome, given the model substrate data using cultured cells above. However, 131 proteins were enriched in the mutant cells, raising the possibility that some changes are mediated by a non-proteolytic manner or through secondary effects, for example, possibly as a part of UPS-autophagy communication (see below).

Our global proteomic analysis was initially validated by comparison with the immunoblotting data on endogenous proteins (Fig. 3d). Consistent with our previous data, levels of proteasome substrates p53 and p62 were both significantly reduced in hyperactive α3ΔN cells, as measured by TMT-MS (Fig. 5c). Degradation of p62, which is subject to both autophagic and proteasomal regulation, appeared to be more directly affected by the hyperactive proteasome. To the contrary, LC3 protein levels were significantly increased, consistent with the increased levels of autophagic selective substrate LC3-II, indicating proteasome activation may negatively regulate autophagy (Figs 3d and 5c, and Supplementary Fig. 2). Accumulating evidence has suggested that the overall activity of UPS affects the autophagy flux in cells: for example, suppression of UPS activity of UPS resulted in induced autophagy^42^,^43^. However, these systems are not communicated by a simple compensatory mechanism in cellular catabolism, because impaired autophagy leads to a decrease of UPS flux, rather than upregulation of UPS activity^43^. The underlying molecular mechanism of this crosstalk is to be determined.

To further validate the legitimacy of the target proteins that are sensitive to hyperactive proteasomes, we used immunoblotting to examine several proteins with significant depletion in the α3ΔN cells from TMT-MS^3^ (Fig. 5d,e). Many target substrates of hyperactive proteasomes identified by quantitative TMT-MS^3^ were validated by immunoblot analysis (Supplementary Fig. 8). For example, proteins whose functions involve cell motion, such as SGPL1, UNC5B, DCDC2, ITGA4, SCARB1 and ApoB, were significantly depleted in α3ΔΝ cells, while levels of proteasome subunits and relative stable proteins, such as α7, ADRM1/RPN13, GAPDH and β-actin, were unchanged (Fig. 5e; Supplementary Fig. 8). Moreover, when comparing the 201 hyperactive proteasome-sensitive substrates with different ubiquitome data sets^44^,^45^,^46^, ∼55% (121 out of 201) of these proteins overlapped between the lists (Supplementary Fig. 9; Supplementary Table 3). These data provide strong evidence that many of the protein targets from our TMT-MS analysis are true substrates of hyperactive proteasomes.

From gene ontology analysis, we identified that a substantial fraction of the hyperactive proteasome targets is enriched in several metabolic and biological processes, including the UPS, protein folding, oxidation/reduction, growth regulation and cellular metabolism (Supplementary Fig. 10; Supplementary Table 4). Further work will be required to determine what distinguishing features of substrates they share to be susceptible to the α3ΔN proteasome. It will be also important to determine the capacity and selectivity of hyperactive proteasomes, especially for the clearance of various proteotoxic proteins.

Next, we examined the levels of various Ub-linkage types, which are crucial determinants of substrate fates. Moreover, different linkages are expected to be regulated and recognized independently although many of related biochemical questions are still unanswered^47^,^48^. We found that, in the α3ΔN cells, the Lys63 (K63)-linked polyubiquitin chains were significantly depleted while most other linkage types were relatively comparable (Fig. 5f; Supplementary Fig. 11; and Supplementary Table 5). Recently, the K63 chain was identified as a novel sensor/regulator of cellular oxidative stress^49^. This result and our previous finding that the hyperactive cells are more resistant to ROS-induced protein oxidation and cytotoxicity (Fig. 4g,h) provide strong evidence that enhanced proteasome activity may relieve oxidative stress from cells. Interestingly, K33-linked polyubiquitin chains, whose biological role has only been studied^50^, were also significantly increased in α3ΔN cells (Fig. 5f). This atypical Ub-linkage type was reported to take only a small portion of the whole ubiquitome in the cell and to be not significantly accumulated after proteasome inhibitor treatments, unlikely other Ub-linkage types^51^. We speculate that K33-linked polyubiquitin chains may function as a sensor of proteasome activity with non-proteolytic consequences, perhaps responding to massive changes of UPS substrates. Collectively, we found that opening the CP gate of proteasomes resulted in global but tolerable proteomic changes in mammalian cells. It has been suggested that proteasomes function under tonic inhibitory states under normal conditions^5^,^52^. Therefore, our proteomic data further indicate enhancing proteasome activity may be a potentially beneficial intervention for cells under mild proteotoxic or oxidative stress.

## Discussion

Here we report that deletion of the α3 subunit's N-terminal tail resulted in activation of mammalian proteasomes, which showed significant increase in hydrolysis of fluorogenic substrate suc-LLVY-AMC and in degradation of Ub-Sic1^PY^ proteins *in vitro*. Opening CP gate enhanced the activity of both free CP and proteasome holoenzymes with translocation-competent conformations, implicating that the gating system may function as a critical regulator of the substrate translocation rates from the RP to the catalytic core. Because the proteasome is a major degradation machinery that regulates the levels of toxic, aggregation-prone proteins and their pathological accumulation^53^, enhancing proteasome activity through gate opening may be beneficial to suppress toxicity and related pathophysiology of proteotoxic diseases, such as Alzheimer's disease^54^,^55^. We observed that cells expressing α3ΔN proteasomes had reduced levels of tau proteins and their aggregates. In addition, mammalian cells with open-gated proteasomes effectively promoted cell survival against ROS-mediated oxidative stress. Considering that CP gate opening was tolerable to cells, the present strategy could be an effective approach to study the regulatory mechanisms of mammalian proteasomes, to identify the molecular link between proteasome activity and autophagic flux, and to modulate the levels of aggregation-prone proteins in the cell. The application of hyperactive proteasomes is actually not limited to neurodegenerative diseases, because numerous other diseases are caused by toxic, misfolded, oxidized, aggregation-prone proteins^56^,^57^. Thus, hyperactive proteasomes with open-gate mutation may have a potentially beneficial effects for cells under various proteotoxic or oxidative stress.

## Methods

### Plasmids

Plasmids expressing α3, α3ΔN, α3-flag and α3ΔN-flag were generated by PCR amplification using specific primers. The PCR products encoding α3 derivatives were digested using restriction endonucleases BamH1 and Xba1. The products were then inserted into the corresponding sites of the pcDNA3.1 plasmid, and digested with the same restriction enzymes to construct the pcDNA3.1-α3 derivatives. The plasmids were then transformed into bacterial strain DH5α to screen for recombinant plasmids. These recombinants were identified by DNA sequencing. Plasmid DNA was prepared and purified using a plasmid midi kit (GeneAll, Korea), according to the manufacturer's instructions, and stored at −20 °C until use. Arg-GFP, RGS4-GFP and LC3-GFP plasmids were previously generated^58^. Vectors expressing tau (from V.M. Lee), α-synuclein (from J.E. Galvin), Ub-Arg-GFPs (from M.G. Masucci) and EGFP-cODC (from P. Coffino) were kindly provided.

### Antibodies and reagents

Sources of antibodies and working dilutions are as follows: anti-tau (clone Tau-5; Invitrogen, USA, 1/10,000); anti-tau^ser396^ (ab109390, Abcam, USA, 1/5,000); anti-tau^ser199/202^ (ab4864, Abcam, 1/5,000); anti-β-actin (A1978, Sigma, 1/10,000); anti-Ub (clone P4D1, Santa Cruz, USA, 1/5,000), anti-Ub conjugates (clone FK2, Enzo Life Science, USA, 1/2,500), anti-Ub (Lys48 specific) (clone apu2, Millipore, USA, 1/2,000), anti-Ub (Lys63 specific) (clone apu3, Millipore, 1/2,000), anti-α3 (PW8115, Enzo Life Science, 1/5,000), anti-α7 (PW8110, Enzo Life Sciences, 1/3,000), anti-ADRM1 (PW9910, Enzo Life Science, 1/2,000), anti-His (A03001, IgTherapy, Korea, 1/2,000), anti-T7 (69522, Millipore, 1/5,000), anti-flag (F1804, Sigma, 1/3,000), anti-Rpt5 (PW8245, Enzo Life Science, 1/3,000), anti-NBR1 (sc130380, Santa Cruz, 1/2,000), anti-p62 (sc28359, Santa Cruz, 1/2,000), anti-p53 (sc1313, Santa Cruz, 1/3,000), anti-LC3 (L7543, Sigma, 1/2,000), anti-USP14 (A300-920A, Bethyl Laboratories, USA, 1/2,000), anti-GFP antibody (Enogene, USA, 1/5,000), anti-LTB4 (bs-5779R, Bioss USA, 1/1,000), anti-FKBP3 (A302-601A, Bethyl, 1/1,000), anti-FKBP4 (A301-426A, Bethyl, 1/1,000), anti-TOR3A (AP17612c, Abgent, 1/1,000), anti-Calnexin (A303-694A, Bethyl, 1/1,000), anti-EPHB2 (bs-0996R, Bioss USA, 1/1,000), anti-ERbB2 (TA503443, OriGene, 1/1,000), anti-ITGB1 (A303-735A, Bethyl, 1/1,000), anti-UNC5B (EB11706, Everest, 1/1,000), anti-TOP2B (C0376, Assay Biotech, 1/1,000), anti-SGPL1 (bs-4188R, Bioss, USA, 1/1,000), anti-DCDC2 (bs-11824R, Bioss, USA, 1/1,000), anti-ITGA4 (4783, ProSci, 1/1,000), anti-ApoB (bs-6333R, Bioss, USA, 1/1,000), anti-SCARB1 (5193, ProSci, 1/1,000), and anti-VAPB (A302-894A, Bethyl, 1/1,000. Sources of major biochemical reagents are as follows: PS-341 (LC Laboratories, USA); epoxomicin and Ub-VS (Boston Biochem, USA); ATP (Calbiochem, USA); ATPγS (Jena Bioscience, Germany); ubiquitin (Sigma); MG132 (Bachem); suc-LLVY-AMC (Bachem); Z-LLE-AMC (Enzo Life Sciences); Boc-LRR-AMC (Enzo Life Sciences). DMEM, FBS and phosphate-buffered saline (PBS) (pH 7.4) were purchased from WelGENE (Korea). CCK-8 (Cell Counting Kit-8) was purchased from Dojindo Molecular Technologies (Japan), and okadaic acid, doxycycline (Dox) and Coomassie Brilliant Blue R250 were purchased from Sigma. EzWay silver staining kit was purchased from Goma Biotech (Korea). Uncropped western blots for each figure are shown in Supplementary Fig. 12.

### Mammalian cell cultures and transient expression

Mammalian cells used in this study, including HEK293, HEK293-pre1-HTBH, HEK293-pre1-HTBH-α3ΔN, HEK293-trex-htau40 and tau-BiFC cells, were grown in DMEM supplemented with 10% FBS, 2 mM glutamine and 100 units ml^−1^ penicillin/streptomycin with frequent mycoplasma tests. Cells were maintained in a humidified incubator with 5% CO_2_ at 37 °C. For transfection, cells were treated with 1–2 μg of total plasmid DNA in a six-well culture plate (>95% confluent or at a density of 10^6^ cells per well) for 36–48 h using Lipofectamine 3000 (Invitrogen). Cell lysates were prepared in RIPA buffer 36–48 h post transfection and were used for immunoblotting. For chase analysis, wild-type and α3ΔN cells were treated with 75 μg ml^−1^ cycloheximide and samples were isolated at chase times 0, 20, 40 and 60 min after 4 h transient MG132 treatment and vigorous washing with PBS. For stable cell line maintenance, transfected cells were cultured with DMEM medium containing 600 mg ml^−1^ G418 and 10% FBS. Fluorescence images were obtained after cells were extensively rinsed three times with PBS.

### RT–PCR

Total RNA from cultured cells was prepared using TRIzol reagent (Invitrogen), followed by further purification through RNeasy mini-columns (Qiagen, USA) with on-column DNase I treatment. cDNA samples were prepared by reverse transcription using Accupower RT-pre mix (Bioneer, Korea). Endogenous α3 was amplified by PCR using forward (5′-ATGTCTCGAAGATATGACTCCAG-3′) and reverse primers (5′-CTATTTATCCTTTTCTTTCTGTTC-3′). Exogenous α3ΔN-flag was amplified using forward (5′-ATGATATTTTCTCCAGAAGGTCGCTTAT-3′) and reverse primers (5′-CTACTTGTCGTCATCGTCTTTGTAGTCTTTA-3′, which is on the C-terminal flag tag). Amplified DNA was visualized by using ethidium bromide after agarose gel electrophoresis.

### Quantitative RT–PCR

Total RNA from cultured cells was prepared using TRIzol reagent (Invitrogen), followed by further purification through RNeasy mini-columns (Qiagen, USA) with on-column DNase I treatment. cDNA samples were prepared by reverse transcription using Accupower RT-pre mix (Bioneer,). Real-time PCR reactions were then performed using the Rotor-Gene RG 3000 system (Corbett Research, Australia) with diluted cDNA, SYBR qPCR master mixture (Kapa Biosystems, USA) as the reporter dye, and 10 pmol of gene-specific primers. Thermal cycling conditions comprised 95 °C for 3 min to allow for enzyme activation, followed by 40 cycles at 95 °C for 10 s, 53 °C for 15 s and 72 °C for 30 s. The level of each mRNA was normalized to that of GAPDH, and the values were plotted as mean±s.d. of three independent experiments. Primer sequences used were as follows: for α3, forward (5-′AGAAGTGGAGCAGTTGATCA-3′) and reverse (5-′TCTCTGATTCTATTTATCCTTTTCT-3′ for endogenous α3, which targets 3′ UTR, or 5-′TCTCTGATTCTACTTGTCGTCATCG-3′ for exogenous α3, which targets the flag tag); for Tau, forward (5′-AAGGTGACCTCCAAGTGTGG-3′) and reverse (5′-GGGACGTGGGTGATATTGTC-3′); for α-Syn, forward (5′-AAGAGGGTGTTCTCTATGTAGGC-3′) and reverse (5′-GCTCCTCCAACATTTGTCACTT-3′); for EGFP forward (5′-ACGTAAACGGCCACAAGTTC-3′) and reverse (5′-AAGTCGTGCTGCTTCATGTG-3′); for GAPDH, forward (5′-GAGTCAACGGATTTGGTCGT-3′) and reverse (5′-GACAAGCTTCCCGTTCTCAG-3′).

### Purification of the 26S human proteasome and α3ΔN-proteasome

Human proteasomes and α3ΔN proteasomes were affinity-purified from a stable HEK293 cell line harbouring biotin-tagged human β4, as previously described, with slight modifications^37^. The cells were cultured in 15-cm culture dishes, collected in lysis buffer (50 mM NaH_2_PO_4_ (pH 7.5), 100 mM NaCl, 10% glycerol, 5 mM MgCl_2_, 0.5% NP-40, 5 mM ATP and 1 mM DTT) containing protease inhibitors, and homogenized using a Dounce homogenizer. After centrifugation, the supernatants were incubated with streptavidin agarose resin (Millipore, Billerica, MA) for 5 h at 4 °C. The beads were washed with lysis buffer and tobacco etch virus buffer (50 mM Tris-HCl (pH 7.5) containing 1 mM ATP and 10% glycerol). The 26S proteasomes were eluted from the resin by incubating with TEV protease (Invitrogen) in TEV buffer containing 1 mM ATP for 1 h at 30 °C and were concentrated using an Amicon ultra-spin column (Millipore).

### Measurement of proteasome activity with fluorogenic peptide substrates

Hydrolysis of fluorogenic substrates suc-LLVY-AMC Boc-LRR-AMC and Z-LLE-AMC was measured to determine the proteolytic activity of the chymotrypsin-like, trypsin-like and caspase-like sites of proteasomes, respectively. For example, a suc-LLVY-AMC hydrolysis assay was carried out using 0.5 nM purified proteasome and 12.5 μM of suc-LLVY-AMC (Enzo Life Sciences). The reaction mixture contained 50 nM Tris-HCl (pH 7.5), 1 mM EDTA, 1 mg ml^−1^ BSA, 1 mM ATP and 1 mM DTT. Proteasome activity, when it is in the engaged conformation, was measured in the presence of 25 nM unmodified or ubiquitinated proteins, and ATPγS was used instead of ATP. Proteasomal activity was monitored by measuring free AMC fluorescence in a black 96-well plate using a TECAN infinite m200 fluorometer.

### *In vitro* ubiquitination of Sic1 and Ub-Sic1 degradation

Polyubiquitinated Sic1 with PY motifs (Ub-Sic1^PY^) was prepared as previously described^29^ with some modifications. Briefly, the Ub conjugation mixture contained 10 pmol Sic1^PY^, 2 pmol Uba1, 5 pmol Ubc4, 5 pmol Rps5 and 1.2 nmol ubiquitin in a buffer of 50 mM Tris-HCl (pH 7.4), 100 mM NaCl, 1 mM DTT, 5 mM ATP and 10 mM MgCl_2_. Conjugation proceeded for 4 h at 25 °C. To purify the conjugates, they were absorbed to a Qiagen Ni-NTA resin, washed with buffer (50 mM Tris-HCl (pH 8.0), 50 mM NaCl and 40% glycerol), eluted with 200 mM imidazole in wash buffer and dialysed into wash buffer containing 10% glycerol. Purified human proteasomes (5 nM) were incubated with 20 nM of Ub-Sic1^PY^ in proteasome assay buffer (50 mM Tris-HCl (pH 7.5), 100 mM NaCl, 10% glycerol, 2 mM ATP, 10 mM MgCl_2_, 1 mM DTT). Ub-Sic1^PY^ degradation was monitored by immunoblotting using an anti-T7 antibody (Millipore).

### Immobilizing proteasomes to nanoparticles

Purified proteasomes and mesoporous silica nanoparticles with nickel moieties (MSNPN) were suspended in PBS, using a variety of indicated molar ratios, and vigorously shaken horizontally for 2 h at room temperature. The resulting proteasome–MSNPN complexes were briefly washed three times by centrifugation at 3,000 r.p.m. The complexes were resuspended in culture media for cellular delivery.

### Assaying tau aggregation in cultured cells

HEK293-trex-htau40 cells were cultured as described above. At ∼60% confluence, the cells were transfected with empty pcDNA 3.1 vector or the vectors containing α3 and α3ΔN insert using LipofectAMINE 3000 transfection reagent (Invitrogen). Cells were treated with 500 ng ml^−1^ Dox for 24 h to induce tau expression after 48 h post transfection, lysed into buffer A (20 mM Tris, pH 7.4, 150 mM NaCl, 1% Triton X-100 and protease inhibitor cocktail), and centrifuged at 200*g* for 15 min at 4 °C. The pellet was collected as P1. The supernatant was further centrifuged at 16,000*g* for 30 min at 4 °C to further separate the Triton X-100-soluble (S2) and -insoluble (P2) fractions. Both P1 and P2 were washed five times with the lysis buffer and resuspended in SDS sample buffer for immunoblotting using anti-tau antibody.

### Tau-BiFC cell analysis

An HEK293-derived stable cell line (Tau-BiFC), which constitutively expresses both the C terminus and the N terminus of Venus protein independently fused with htau40 (ref. 39). Tau-BiFC cells were seeded in a 96-well plate at a density of 10^5^ cells per well and were transfected with plasmid for 24 h. Then, 30 nM of okadaic acid was added for 24 h to accelerate the tau oligomerization processes. Fluorescence images were quantified using Image J software (ver. 1.48k, NIH).

### Assessment of cell viability

Cell viability was assessed using a modified MTT assay. HEK293-based stable cells were treated with menadione at various concentrations (5–25 μM) for 4 h, followed by the addition of 10 ml of 5 mg ml^−1^ thiazolyl blue tetrazolium bromide (MTT, Sigma) solution to the media and incubation for 2.5 h at 37 °C in a humidified atmosphere of 95% air and 5% CO_2_. After discarding the media, 200 ml DMSO was added to solubilize the blue MTT-formazan product, and the cells were incubated for an additional 30 min at room temperature. The absorbance of the solution was read at 570 nm (test) and 630 nm (reference).

### Oxidized protein assays

Oxidized proteins were detected using the OxyBlot protein oxidation detection kit (Millipore). Briefly, total proteins from cells were isolated after treatment with 25 μM of menadione for 2 h, and 15 μg of protein was used for derivatization with 2-4-dinitrophenyl hydrazine for 25 min. Samples were resolved by SDS–PAGE and anti-DNP antibody was used for subsequent immunoblotting.

### Mass spectrometry analysis

Protein samples were prepared from wild-type and α3ΔN cells in three separate 150 mm dishes. Cells were washed three times with ice-cold PBS, then scraped in PBS, spun down and lysed in 8 M urea lysis buffer (8 M urea, 75 mM NaCl, 50 mM HEPES pH 8.0, with added Complete protease inhibitors (Roche) and PhosSTOP phosphatase inhibitors (Roche)). Cell debris was spun down for 10 min at 13,000 r.p.m. at 4 °C, after which protein concentrations were determined using the BCA assay (Thermo Fisher Scientific). Subsequently, 400 μg of lysate was reduced with 5 mM TCEP (tris(2-carboxyethyl)phosphine) for 30 min and alkylated with 14 mM iodoacetamide for 30 min in the dark. Proteins were precipitated using methanol/chloroform precipitation and resuspended in digestion buffer (8 M urea, 50 mM HEPES pH 8.5 and 1 mM CaCl_2_). The protein extracts were diluted to 4 M urea, after which they were digested for 2 h at 37 °C with LysC (Wako) at a 1:250 LysC/protein ratio. They were then further diluted to 2 M urea, and incubated overnight at 37 °C with LysC. The next day, urea was further diluted to 1 M and trypsin (Promega) was added at a 1:50 trypsin/protein ratio for 6 h at 37 °C. The samples were acidified with formic acid (FA) to a pH of <2, and then desalted using Sep-Pak C18 solid-phase extraction cartridges (Waters). Peptide concentrations were determined using the micro-BCA assay (Thermo Fisher Scientific), after which the samples were labelled with the 6-plex TMT reagents (Thermo Fisher Scientific). TMT labelling and subsequent MS analysis were performed largely as described previously^59^. Briefly, 0.8 mg of TMT reagents was dissolved in 40 μl anhydrous acetonitrile (ACN) and 10 μl was added to 100 μg peptides in 90 μl of 200 mM HEPES, pH 8.5. After 2 h, the reaction was quenched with 8 μl of 5% hydroxylamine (Sigma). Labelled peptides were combined at a ratio of 1:1:1:1:1:1 for the six channels, acidified with FA, diluted to a final concentration of 3% ACN, and then desalted with a Sep-Pak column. The peptides were then subjected to basic-pH reverse-phase HPLC fractionation as described^60^ and fractionated into 24 fractions. Half of these fractions were dissolved in 3% FA/3% ACN, desalted via StageTip, dried in a SpeedVac, and then dissolved in 8 μl of 3% FA/3% ACN for LC-MS/MS analysis on an Orbitrap Fusion mass spectrometer (Thermo Fisher Scientific) as described previously^61^. Briefly, peptides were separated on an in-house packed column using a gradient of 85 min from 6 to 24% ACN in 0.125% FA at 575 nl per minute. FTMS1 spectra were collected at a resolution of 120k with a maximum injection time of 100 ms and a 200k automated gain control (AGC) target. A top-10 method was used to select the 10 most intense ions for MS/MS. ITMS2 spectra were collected with a maximum injection time of 150 ms with an AGC target of 4k and CID collision energy of 35%. FTMS3 spectra were collected using the multi-notch method described previously^59^ to reduce interference and to increase quantitative sensitivity and accuracy. In brief, synchronous-precursor selection was used to include 10 MS2 fragment ions in the FTMS3 scan. To create TMT reporter ions, the higher-energy collisional dissociation collision energy was set at 55%. An AGC target of 50k and maximum injection time of 250 ms were used. Mass spectra were processed using an in-house software pipeline as described previously^60^. In short, mass spectra were searched against the human Uniprot database (February 2014) and a reverse decoy database. Precursor ion tolerance was set at 20 p.p.m. and product ion tolerance at 0.9 Da. Addition of a TMT tag (+229.1629 Da) on lysine residues and peptide N-termini, and cysteine carbamidomethylation (+57.0215 Da) were added as static modifications, and methionine oxidation (+15.9949 Da) was set as a variable modification. A separate search was done for the ubiquitin linkages, in which a differential modification of +114.0429 Da for the GG-peptide on lysine residues was added. False discovery rate was set at 1%, and peptide spectral match filtering was performed using linear discriminant analysis as described previously^60^. After exporting the protein quantification values, the data was further analysed in Excel and Perseus 1.5.1.6. For protein quantitation, the signal-to-noise values for each reporter ion channel were summed across all quantified peptides, and then normalized assuming equal peptide loading across all samples. A two-tailed *t*-test was then performed to identify significantly changed proteins between the wild-type and α3ΔN cells triplicates, after which the *P* values were corrected for multiple testing using the Benjamini–Hochberg method^62^. For the ubiquitin linkage searches, GG-sites were localized using a modified version of the Ascore algorithm^63^, using a localization threshold of 13. The relative ubiquitin linkage abundance was determined by normalizing the quantified linkage-specific peptide to the amount of total ubiquitin in each channel. Gene ontology analysis was performed using the DAVID Bioinformatics Resource 6.7 functional annotation tool (http://david.abcc.ncifcrf.gov/)^64^,^65^. The mass spectrometry proteomics data have been deposited to the ProteomeXchange Consortium via the PRIDE^66^ partner repository with the data set identifier PXD003577.

### Statistical analysis

Statistical significance of difference between various groups was determined by one-way analysis of variance followed by the Bonferroni *post hoc* test in most data. Differences were considered to be significant *P*<0.05. The Michaelis–Menten kinetic parameters were obtained by fitting the experimental data to a nonlinear regression model, using GraphPad Prism 5 (GraphPad Inc.).

## Additional information

**Accession code:** Proteomic raw data are available via ProteomeXchange with identifier PXD003577.

**How to cite this article:** Choi, W. H. *et al.* Open-gate mutants of the mammalian proteasome show enhanced ubiquitin-conjugate degradation. *Nat. Commun.* 7:10963 doi: 10.1038/ncomms10963 (2016).

## Supplementary Material

Supplementary Information Supplementary Figures 1-12

Supplementary Table 1 Quantified proteins from wt and a3DN cells

Supplementary Table 2 a3DN proteasome targets (>2-fold change, p<0.05)

Supplementary Table 3 Comparison with ubiquitomes

Supplementary Table 4 GO enrichment by DAVID functional annotation tool

Supplementary Table 5 Relative Ub linkage abundance
