# Supplementary material for: Open-gate mutants of the mammalian proteasome show enhanced ubiquitin-conjugate degradation
Source: Nat Commun. 2016 Mar 9;7:10963. doi: 10.1038/ncomms10963 (PMC4786872; doi:10.1038/ncomms10963)
Supplement: Supplementary Information — Supplementary Figures 1-12 [file ncomms10963-s1.pdf]

Supplementary Figures

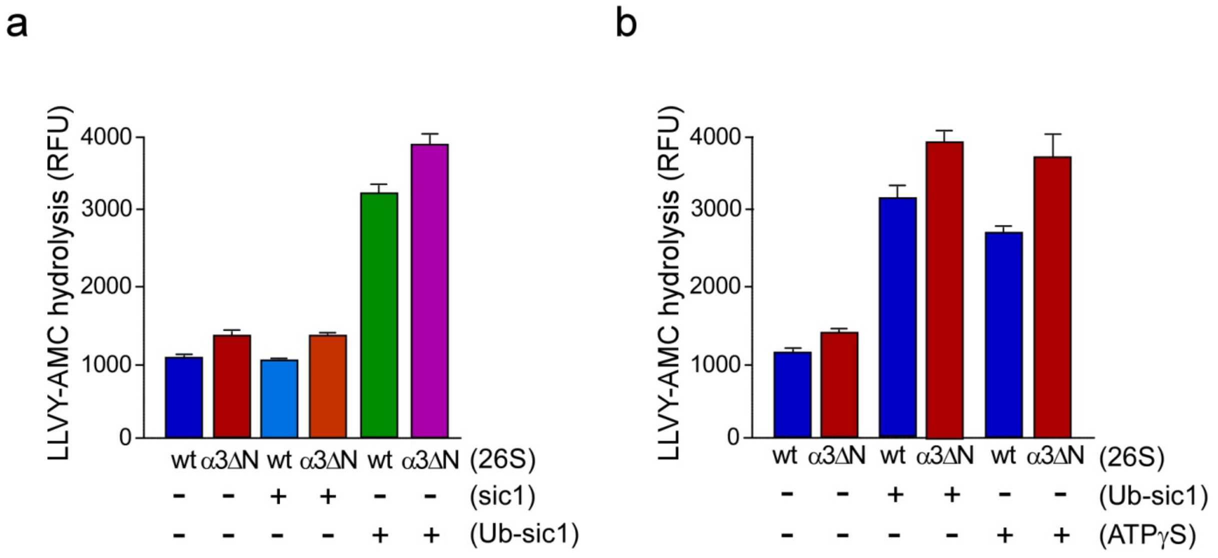

Supplementary Figure 1. Activation of wild-type (wt) and α3ΔN 26S proteasomes by polyubiquitinated proteasome substrates (**a**) and ATPγS (**b**). Purified 26S proteasomes, both wt and α3ΔN, showed significantly higher suc-LLVY-AMC hydrolysis activity in the presence of polyubiquitinated Sic1 (Ub-Sic1^{PY}) proteins or ATPγS, but not by non-ubiquitinated Sic1 proteins. The activation of 26S proteasome reflects the conformational changes of the RP from non-engaged to engaged, translocation-competent states.

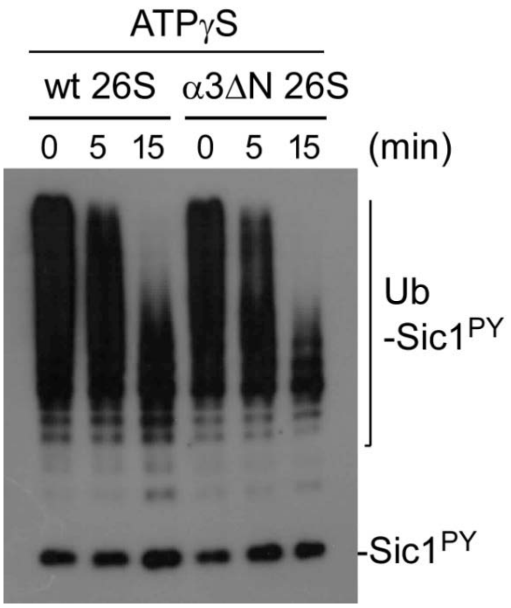

Supplementary Figure 2. ATPγS-enriched conditions delayed *in vitro* Ub-Sic1^{PY} degradation by purified 26S proteasomes. However, α3ΔN 26S proteasomes still showed significantly facilitated Ub-Sic1^{PY} degradation. Note that the disappearance of slowly migrating Ub-Sic1 species are mainly due to the deubiquitination of Ub-Sic1 to Sic1 (indicated by Sic1^{PY}), possibly by USP14 on the proteasome.

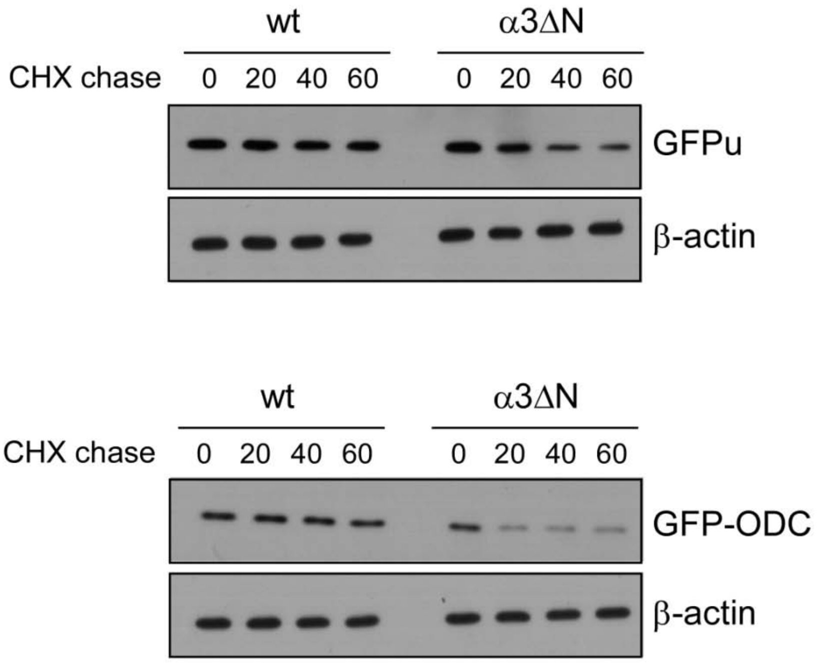

Supplementary Figure 3. Accelerated substrate degradation by α3ΔN gate opening. The ubiquitin-dependent proteasome substrate GFPu and the ubiquitin-independent GFP-ODC proteins were transiently overexpressed in wt and α3ΔN cell lines. Cells were pre-treated with MG132 (20 μM) for 4 h and extensively washed with PBS. Note that the levels of proteasome substrates were highly comparable at time zero. Then chase experiments were carried out at the indicated time points after the addition of 75 μg/mL cycloheximide (CHX) at time zero. Anti-GFP and anti-β-actin antibodies were simultaneously incubated for immunoblotting. These data complement Fig. 3c, where the quantification was performed using ImageJ software (ver. 1.48k, NIH).

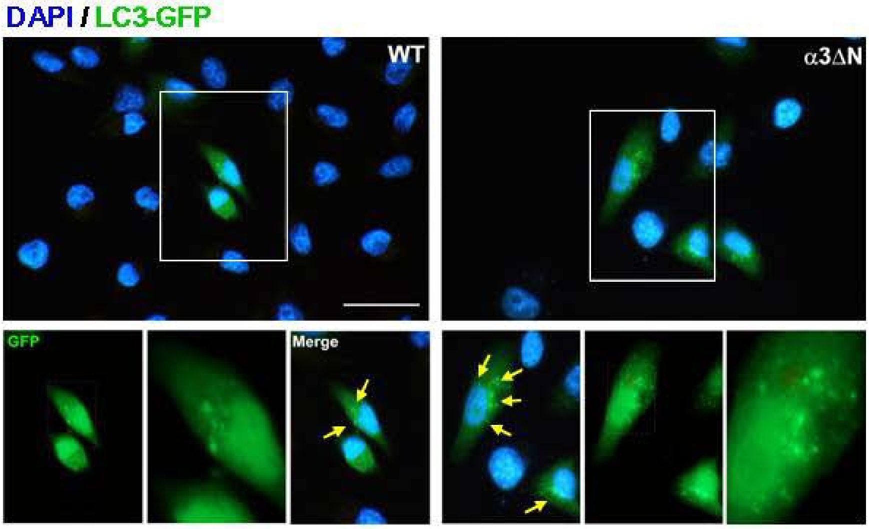

Supplementary Figure 4. Microscopic fluorescence analysis using LC3-GFP and DAPI counterstaining in wt and α3ΔN cell lines. Arrows indicate LC3 puncta in the cells. These data complement Fig. 3d.

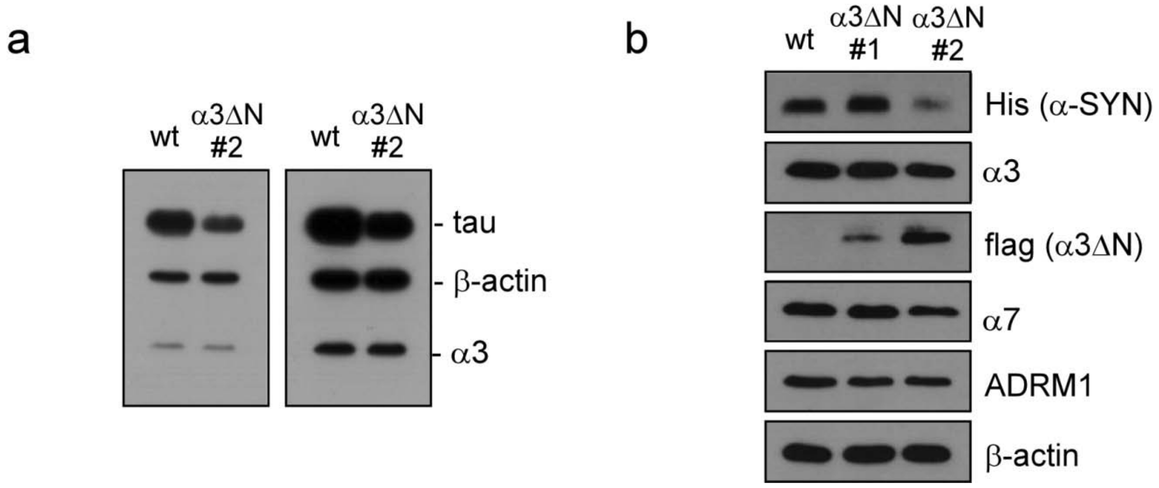

Supplementary Figure 5. Hyperactive α3ΔN proteasomes enhances degradation of tau (**a**) and α-synuclein (α-SYN) (**b**). These proteins are transiently overexpressed in wt and α3ΔN cells for 24 h, followed by immunoblotting analysis for various proteasome subunits and β-actin. These data complement Fig. 3f and Fig. 3g.

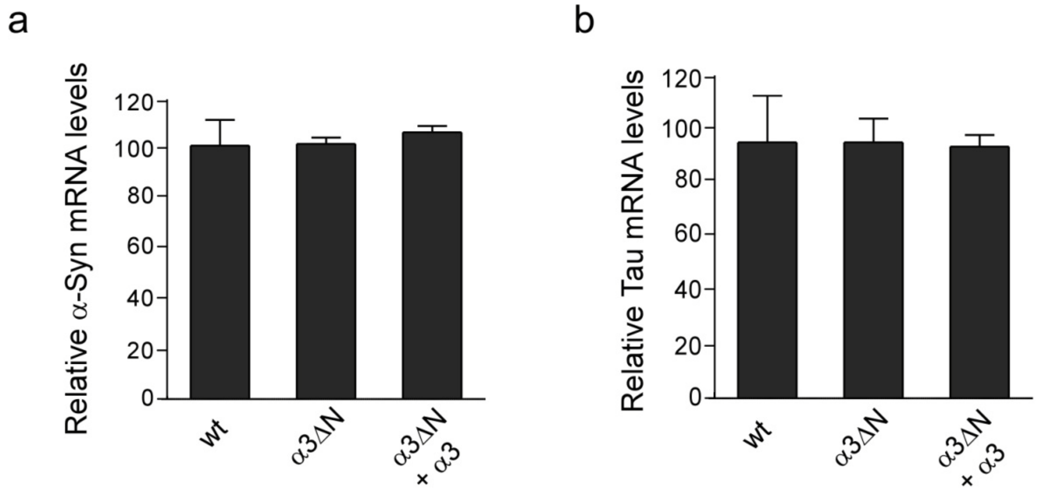

Supplementary Figure 6. Post-translational α-SYN and tau regulation by open-gated mutant proteasomes. qRT-PCR was performed using primers for α-Syn (**a**) and tau (**b**) and GAPDH (control for normalization). The values plotted are the means ± SD of three independent experiments (n=3). These data also complement Fig. 3f and Fig. 3g.

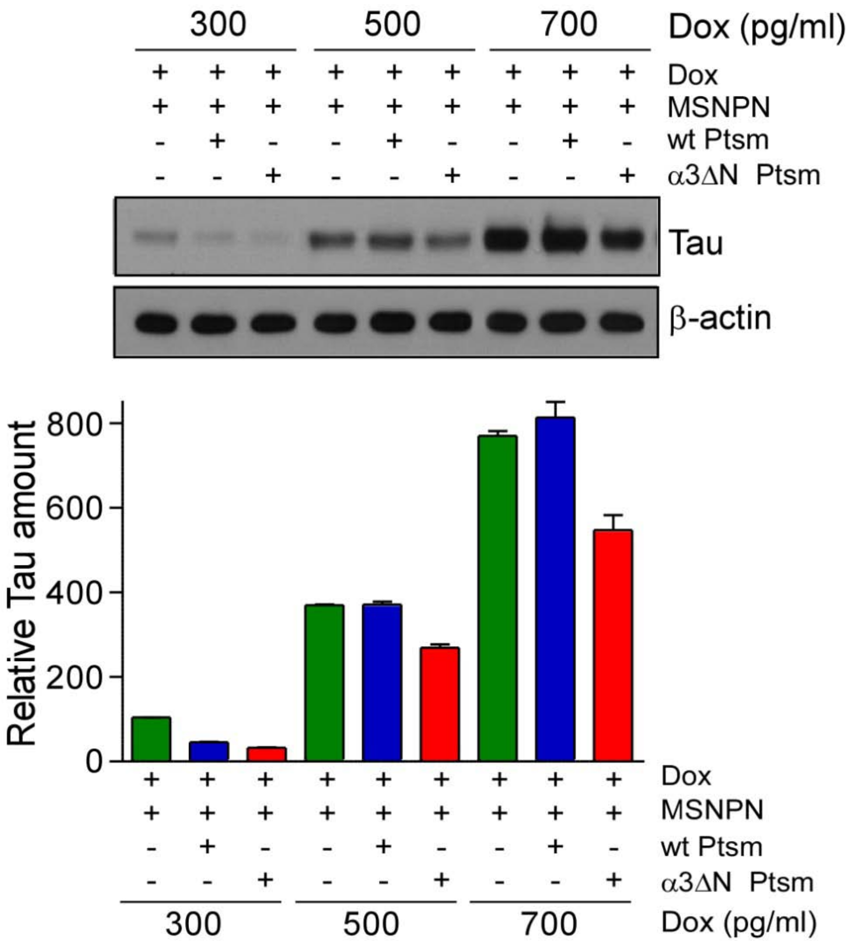

Supplementary Figure 7. Direct cellular delivery of purified wild-type (wt) and α3ΔN mutant proteasomes using Ni^{2+}-tagged mesoporous silica-nanoparticles. Tau proteins were induced in HeLa cells using doxycyline (Dox) with the indicated concentrations.

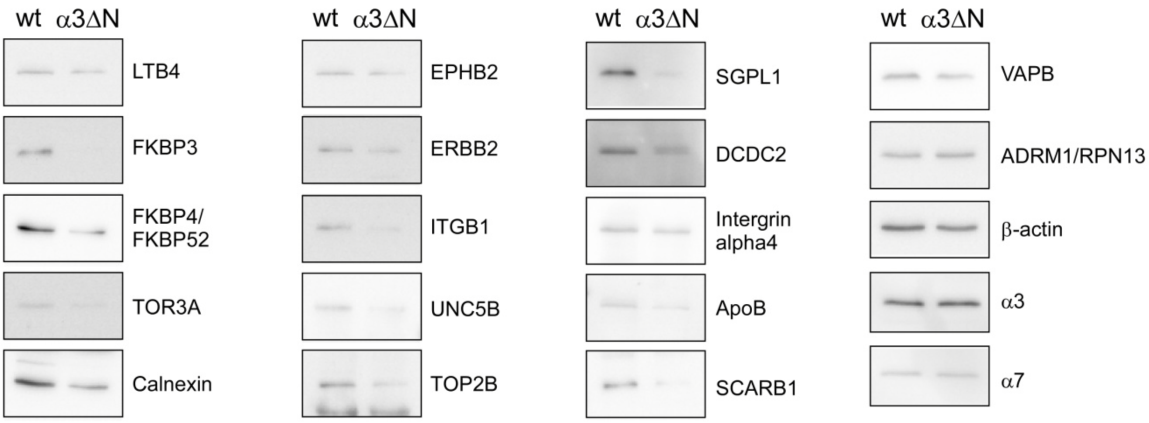

Supplementary Figure 8. Immunoblotting analysis of candidate targets identified from TMT-MS.

a

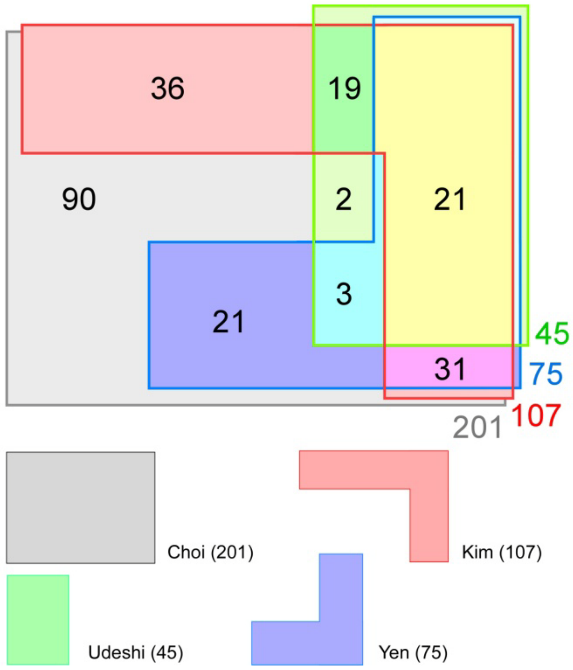

b

Gene Symbol	Description
STAT1	Signal transducer and activator of transcription 1-alpha/beta
ATP2A2	Sarcoplasmic/endoplasmic reticulum calcium ATPase 2
CANX	Calnexin
CANX (fragment)	Calnexin (fragment)
CD47	Leukocyte surface antigen CD47
COMT	Catechol O-methyltransferase
DHCR7	7-dehydrocholesterol reductase
FDFT1	Squalene synthase
HLA-A	HLA class I histocompatibility antigen, A-2 alpha chain
HLA-C	HLA class I histocompatibility antigen, Cw-7 alpha chain
ITM2B	Integral membrane protein 2B
ITM2C	Integral membrane protein 2C
LCP1	Plastin-2
PFKP	6-phosphofructokinase type C
RDH11	Retinol dehydrogenase 11
SLC20A1	Sodium-dependent phosphate transporter 1
SLC29A1	Equilibrative nucleoside transporter 1
SLC38A2	Sodium-coupled neutral amino acid transporter 2
SPINT2	Kunitz-type protease inhibitor 2
TBL2	Transducin beta-like protein 2
TM7SF3	Transmembrane 7 superfamily member 3

Supplementary Figure 9. Comparison with ubiquitomes generated from different studies. (**a**) Venn diagram showing numbers of shared proteins among ubiquitome data sets, which include this study, Kim *et al* (Molecular Cell, 2011, *44*, 325-340), Yen *et al* (Science, 2008, *322*, 918-923), and Udeshi et al (Nature Protocol, 2013, *8*, 1950-1960). (**b**) List of 21 proteins commonly found in all ubiquitome data sets (yellow shades of panel **a**).

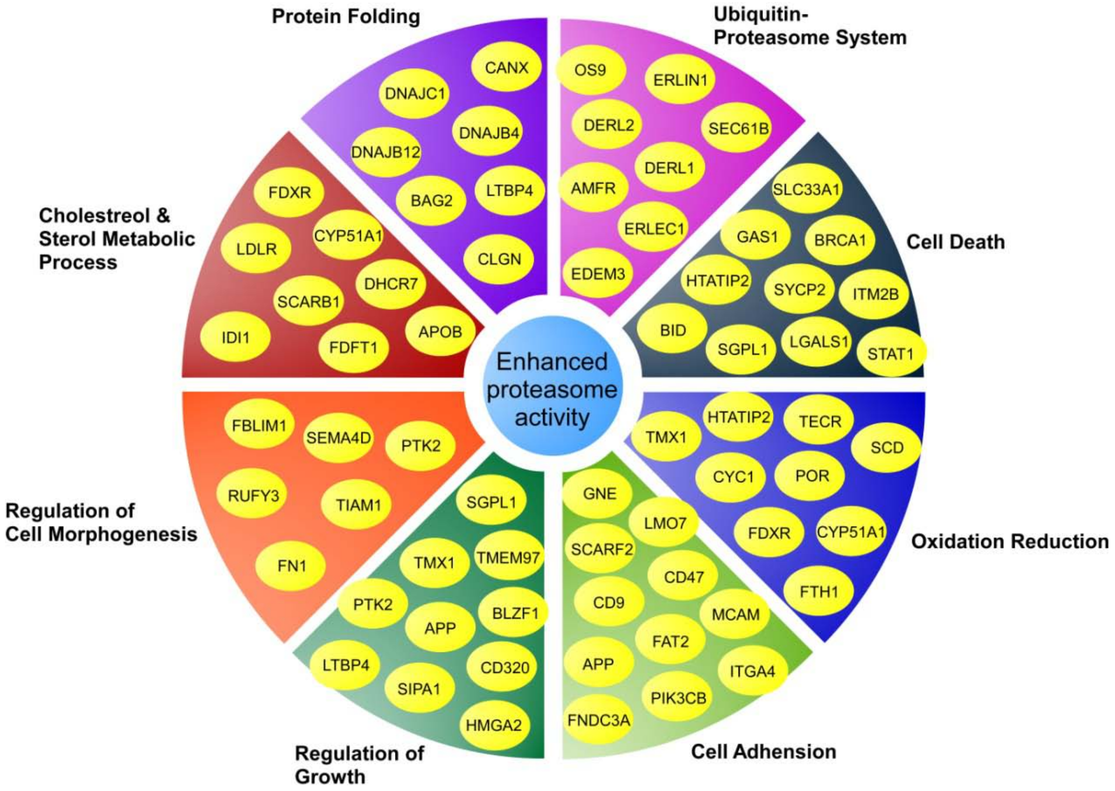

Supplementary Figure 10. Highlights of several important biological pathways and functions enriched from target proteins identified in Supplementary Table 2.

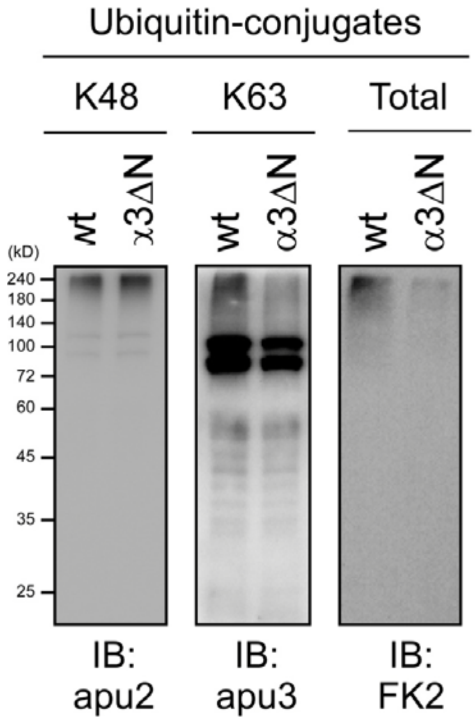

Supplementary Figure 11. Comparing K48- and K63-linked Ub chains in wt and α3ΔN cells. Immunoblotting analysis of whole cell extracts using monoclonal antibodies specific for Lys48 linkage (apu2 clone), Lys63 linkage (apu3 clone), and total Ub conjugates (FK2 clone). These data complement Fig. 5f and Supplementary Table 5.

a

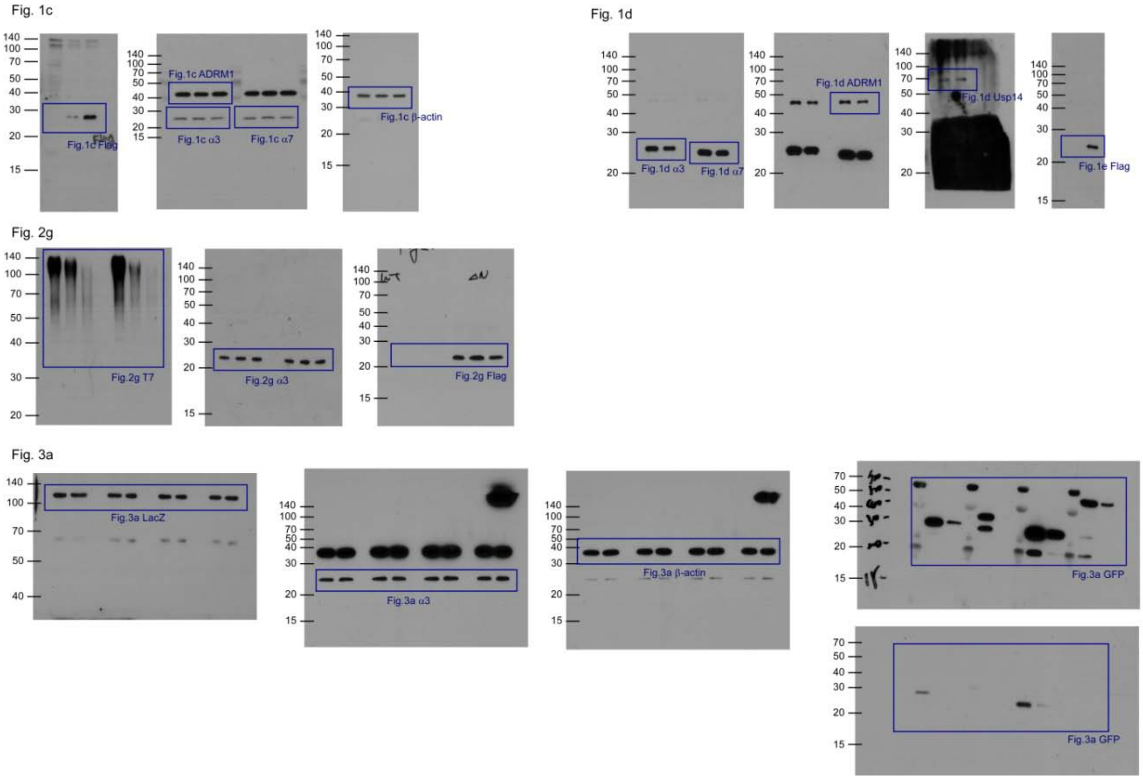
Fig. 1c
Fig.1c Flag
Fig.1c ADRM1
Fig.1c α3
Fig.1c α7
Fig.1c β-actin
Fig. 1d
Fig.1d α3
Fig.1d α7
Fig.1d ADRM1
Fig.1d Usp14
Fig.1e Flag
Fig. 2g
Fig.2g T7
Fig.2g α3
Fig.2g Flag
Fig. 3a
Fig.3a LacZ
Fig.3a α3
Fig.3a β-actin
Fig.3a GFP
Fig.3a GFP

b

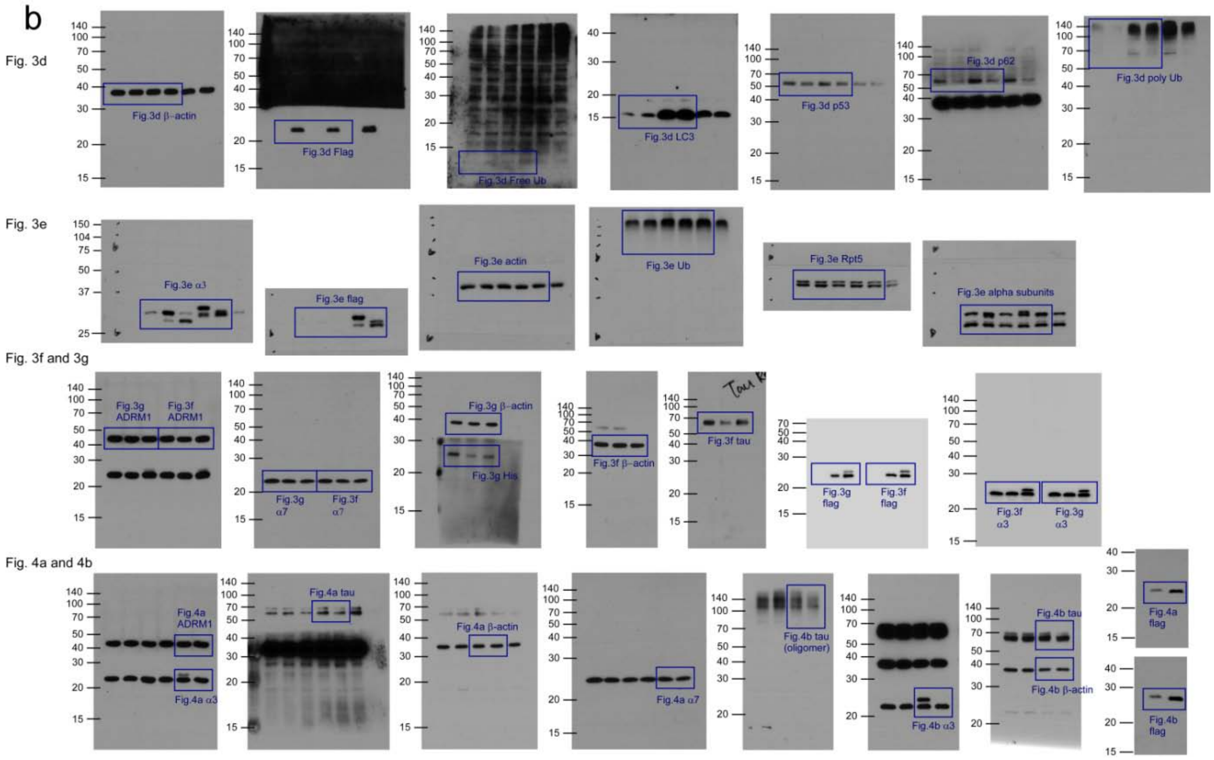
Fig. 3d
Fig.3d β-actin
Fig.3d Flag
Fig.3d Free Ub
Fig.3d LC3
Fig.3d p53
Fig.3d p62
Fig.3d poly Ub
Fig. 3e
Fig.3e α3
Fig.3e flag
Fig.3e actin
Fig.3e Ub
Fig.3e Rpt5
Fig.3e alpha subunits
Fig. 3f and 3g
Fig.3g ADRM1
Fig.3f ADRM1
Fig.3g α7
Fig.3f α7
Fig.3g β-actin
Fig.3g His
Fig.3f β-actin
Fig.3f tau
Fig.3g flag
Fig.3f flag
Fig.3f α3
Fig.3g α3
Fig. 4a and 4b
Fig.4a ADRM1
Fig.4a α3
Fig.4a tau
Fig.4a β-actin
Fig.4a α7
Fig.4b tau (oligomer)
Fig.4b α3
Fig.4b tau
Fig.4b β-actin
Fig.4a flag
Fig.4b flag

c

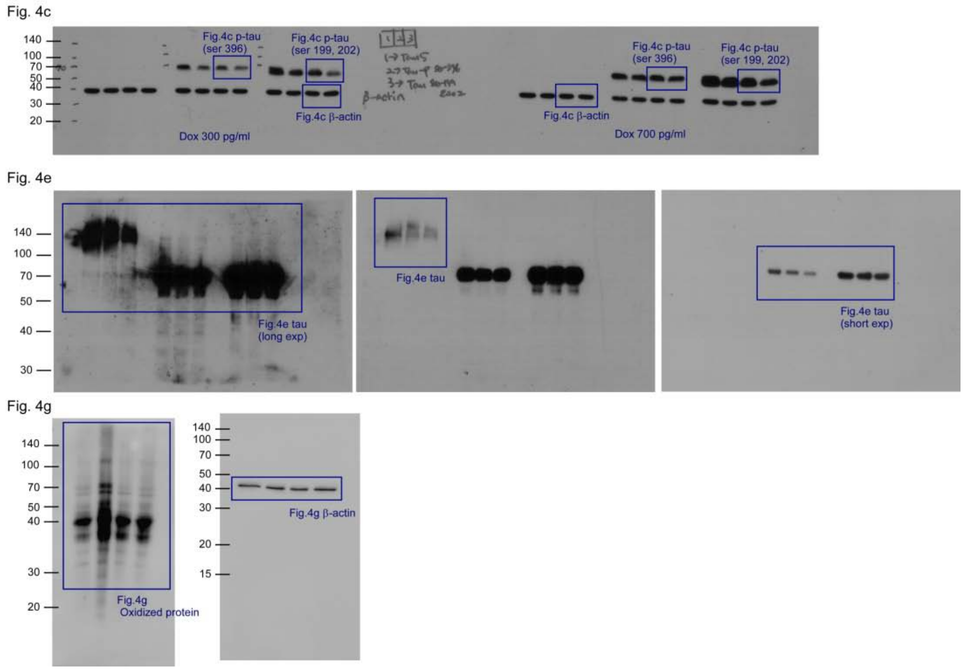

d

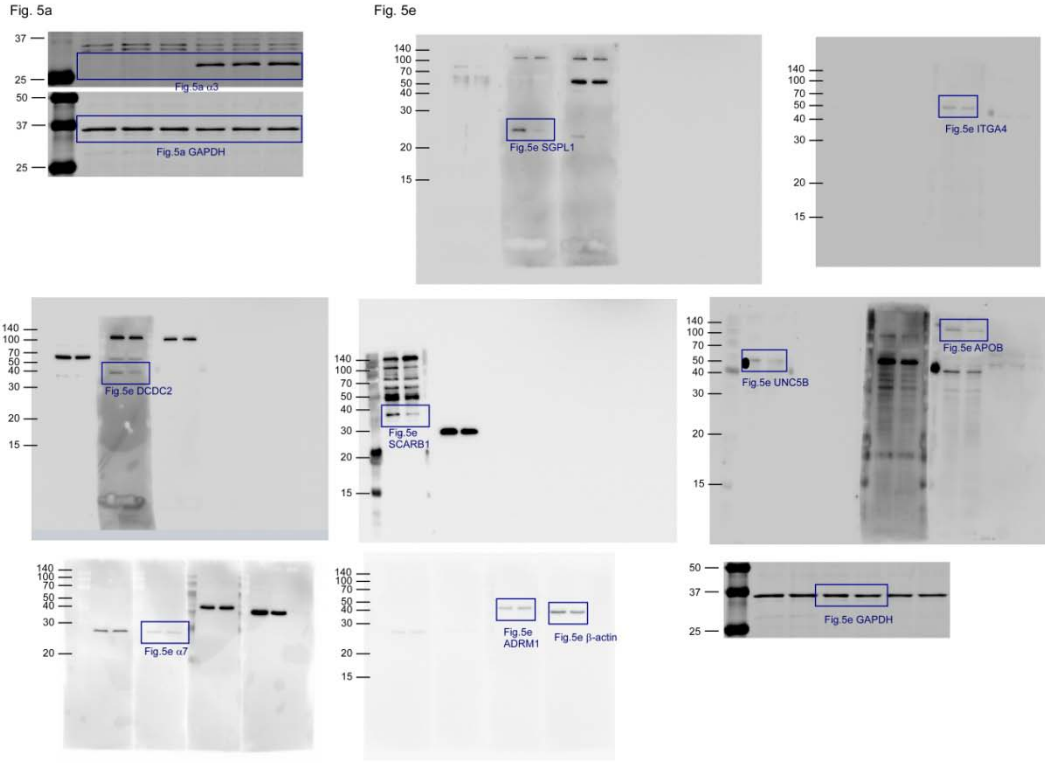

Supplementary Figure 12. Full-size scans of original immunoblotting membranes are shown for the corresponding panels.
